# Correlated Mutation Analysis on the Catalytic Domains of Serine/Threonine Protein Kinases

**DOI:** 10.1371/journal.pone.0005913

**Published:** 2009-06-15

**Authors:** Feng Xu, Pan Du, Hongbo Shen, Hairong Hu, Qi Wu, Jun Xie, Long Yu

**Affiliations:** 1 State Key Laboratory of Genetic Engineering, Institute of Genetics, School of Life Sciences, Fudan University, Shanghai, China; 2 Institute of Biomedical Sciences, Fudan University, Shanghai, China; 3 Biomedical Informatics Center, Northwestern University, Chicago, Illinois, United States of America; Griffith University, Australia

## Abstract

**Background:**

Protein kinases (PKs) have emerged as the largest family of signaling proteins in eukaryotic cells and are involved in every aspect of cellular regulation. Great progresses have been made in understanding the mechanisms of PKs phosphorylating their substrates, but the detailed mechanisms, by which PKs ensure their substrate specificity with their structurally conserved catalytic domains, still have not been adequately understood. Correlated mutation analysis based on large sets of diverse sequence data may provide new insights into this question.

**Methodology/Principal Findings:**

Statistical coupling, residue correlation and mutual information analyses along with clustering were applied to analyze the structure-based multiple sequence alignment of the catalytic domains of the Ser/Thr PK family. Two clusters of highly coupled sites were identified. Mapping these positions onto the 3D structure of PK catalytic domain showed that these two groups of positions form two physically close networks. We named these two networks as θ-shaped and γ-shaped networks, respectively.

**Conclusions/Significance:**

The θ-shaped network links the active site cleft and the substrate binding regions, and might participate in PKs recognizing and interacting with their substrates. The γ-shaped network is mainly situated in one side of substrate binding regions, linking the activation loop and the substrate binding regions. It might play a role in supporting the activation loop and substrate binding regions before catalysis, and participate in product releasing after phosphoryl transfer. Our results exhibit significant correlations with experimental observations, and can be used as a guide to further experimental and theoretical studies on the mechanisms of PKs interacting with their substrates.

## Introduction

Phosphorylation of protein substrates by PKs is the most abundant and important type of cellular regulation [1]. In eukaryotes, PKs mainly phosphorylate serine and threonine residues (Ser/Thr PKs) or tyrosine residues (Tyr PKs). The vast majority of PKs are Ser/Thr PKs. Previous studies [2], [3] on PK structures have shown that the basic fold of the catalytic domains of PKs is structurally well conserved (i.e., two-lobe structure), and the peptide substrates are always held in the groove between the two lobes in many PK-substrate complex structures. Despite these highly conserved characteristics of the catalytic domains, different PKs recognize different consensus sequences in their substrates [2], [4]. It has been estimated that about 30% of all cellular proteins are phosphorylated on at least one residue [4], [5]. Great progresses have been made in understanding the mechanisms of PKs phosphorylating their proper substrates [6], [7]. These mechanisms include the structure of catalytic cleft, consensus sequences, local and distal interactions between kinase and substrate. However, the detailed mechanisms, by which the structurally conserved catalytic domains of PKs recognize and interact with their diverse substrates, still have not been adequately understood.

Thanks to the availability of large sets of diverse sequences [8], we can apply correlated mutation analysis to study Ser/Thr PKs. Correlated mutation analysis serves as the most promising approach and it has been widely used for predicting covariant sites in proteins, which often contain the information of intra-molecular or inter-molecular contacts [9], [10]. Such covariance is usually inferred from statistical analysis among the members of the examined protein family. Statistical coupling analysis (SCA), mutual information (MI) analysis and residue correlation analysis (RCA) are the most typical covariance analysis algorithms. These methods have been used to detect inter-residue contacts within proteins [11]–[13], identify communication pathways in allosteric proteins [14]–[18] and study drug-induced mutations using clinical data [19], [20]. No single method has proved itself vastly superior to others [21] and different methods have different sensitivities to identical background conservation [22]. In recent years, some modifications over these methods were proposed in an attempt to reduce the proportion of false-positive predictions [23]–[26]. However, relatively few studies have systematically evaluated the degree to which these modifications are superior to the original methods. However, by combining multiple methods to compensate each other, it is possible to get more reliable results [27].

In this article, three covariance analysis algorithms were separately applied to analyze a structure-based MSA of the catalytic domains of Ser/Thr PKs. We identified two distinct groups of highly coupled amino acid positions in the catalytic domains by combining the results of these three methods. Mapping these positions onto the typical two-lobe structure of PK catalytic domain showed that these two groups of positions formed two different physically close networks: θ-shaped and γ-shaped networks. The θ-shaped network links the active site cleft and substrate binding regions. This network might participate in PKs recognizing and interacting with their substrates. The γ-shaped network, linking the activation loop and substrate binding regions, might play an important role in supporting the activation loop and substrate binding regions before catalysis, and participate in product releasing after catalysis. Some of the residues identified in these two networks have been shown to be important in interacting with substrates. Our results can provide some new insights into the mechanisms of interactions between PKs and substrates.

## Results

### Sequence Collection and Pretreatment

In order to ensure that sequences of alignment are representative and diverse, we collected the homologue sequences by using seventeen different sequences as initial query sequences. These initial query sequences come from nine eukaryotic organisms including vertebrate, invertebrate, plant and fungus, and they are distributed over all subfamilies of Ser/Thr PKs (See Supporting Information Figure S1) [8], [28]. These collected homologue sequences are from 206 eukaryotic organisms, and therefore can adequately represent the properties of the Ser/Thr PK catalytic domain family and eliminate the phylogenetic bias in the collection of sequences. For the sake of obtaining high quality alignments, we used a structure-based sequence alignment server, FUGUE [29], to align our collected sequences. It has been known that protein structure is far more conserved than protein sequence over the course of evolution [30], so we can extract more evolutionary information from a structure-based MSA. To avoid apparent co-variation due to a common phylogenetic origin of closely related sequences, any redundant sequences must be removed [14], [31]. This was accomplished by creating a new alignment and adding one sequence at a time from the old alignment, where sequences were added only if they had <90% identity to all sequences already in the new alignment. At last, the new alignment has 1112 sequences, and this alignment becomes the basis for the following analyses. The partial sequence alignment result is showed in Supporting Information Figure S2.

The alignment generated many gaps. According to previous studies [27], the number of gaps in each column has influence in the coupling energy calculation of SCA. To reduce this potential influence, we removed the columns with more than 47 gaps (>5%) in the alignment. This resulted in an 1112×223 matrix, which was used for RCA, MI analysis and the calculation of coupling energy of SCA. This alignment is available for download in Supporting Information Text S1.

### Statistical Coupling and Mutual Information Analyses

**Figure 1:**
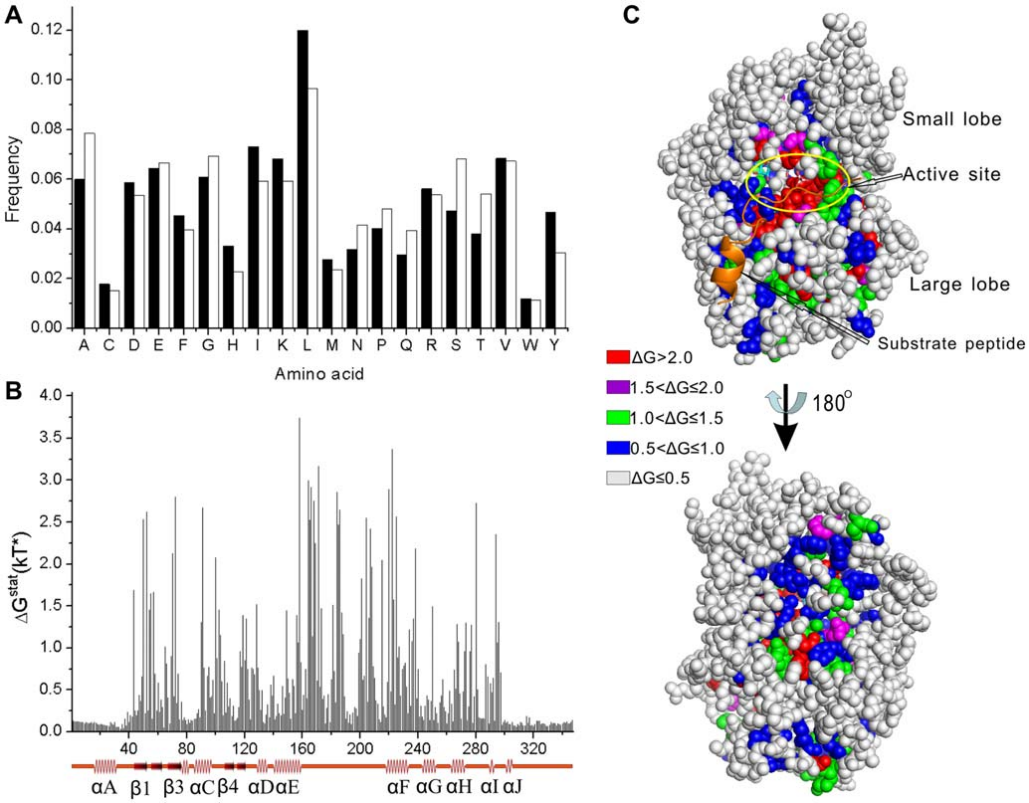
Amino acid frequencies and static energy. (A) A comparison of amino acid distributions in the MSA (filled bars) and in all proteins from the Swiss-Prot database (open bars). (B) The static energy (arbitrary unit) is plotted against primary structure using the numbering of PKAc. The main α-helices and β-sheets are shown below the corresponding positions. (C) A mapping of static energy onto the structure of PKAc (1ATP). The orange cartoon model shows the peptide ligand of PKA. The stick model represents ATP molecule bound at active site cleft of PKAc.

To find out whether our MSA is a well-sampled set for the Ser/Thr PK catalytic domain family, we first compared the overall amino acid distributions of MSA with those of all proteins from the Swiss-Prot database [32]. Note that due to the large sequence divergence of this family, there are only slight differences between the overall amino acid distributions of all proteins and those of Ser/Thr PK catalytic domains alone (Figure 1A), which indicates that the collected sequences are representative of the Ser/Thr PK catalytic domain family, and amino acid distributions at sites are indeed reflective of the functional or structural constraints on this family [14], [18]. In addition, the MSA should be so large that random elimination of sequences from the alignment will not change the amino acid conservation at each site. We tested our MSA following the methods described in [18] and concluded that our MSA is large enough and satisfies the condition of statistical equilibrium (see Supporting Information Figure S3A). The magnitude of the static energy represents the extent of deviation of the amino acid distribution at each site from the mean of the alignment, and therefore it represents the extent of residue conservation at that site. Figure 1B shows the static energy of all 347 positions using the numbering of catalytic domain of cAMP-dependent protein kinase (PKAc). This figure describes the overall conservation profile of the Ser/Thr PK catalytic domain family. The static energy for all positions was then mapped onto the structural model of PKAc (Figure 1C). The most conserved positions are mainly located in the active site cleft, and the intermediately conserved positions are clustered in other functionally important regions. This finding is consistent with the intuitive expectation that a proper measure of conservation should be able to map functionally important sites on a protein.

At the second step, we performed perturbations to calculate the statistical coupling energy [14], [18]. There are 126 sites with at least one moderately conserved residue (30%<p<85%). Similarly, perturbations at sites in the MSA should produce sub-alignments that are also large and diverse enough so that they still represent subsets of the parent MSA and do not substantially alter the state of statistical equilibrium. We used a method similar with the above to test each perturbation site, and we found that all 126 perturbation sites used by us satisfy the criterion of statistical equilibrium (Figure S3B). Perturbations were performed (one perturbation at each site) for these 126 sites, and 126 subsets were generated. The extracted sequences in a subset containing only the most conserved residue at the perturbation site resulted in amino acid redistribution at this and all other sites. If the perturbation at one site significantly changes the amino acid distribution at another site, then these two sites have high coupling energy. Otherwise, they have low coupling energy. This resulted in a 223×126 matrix.

**Figure 2:**
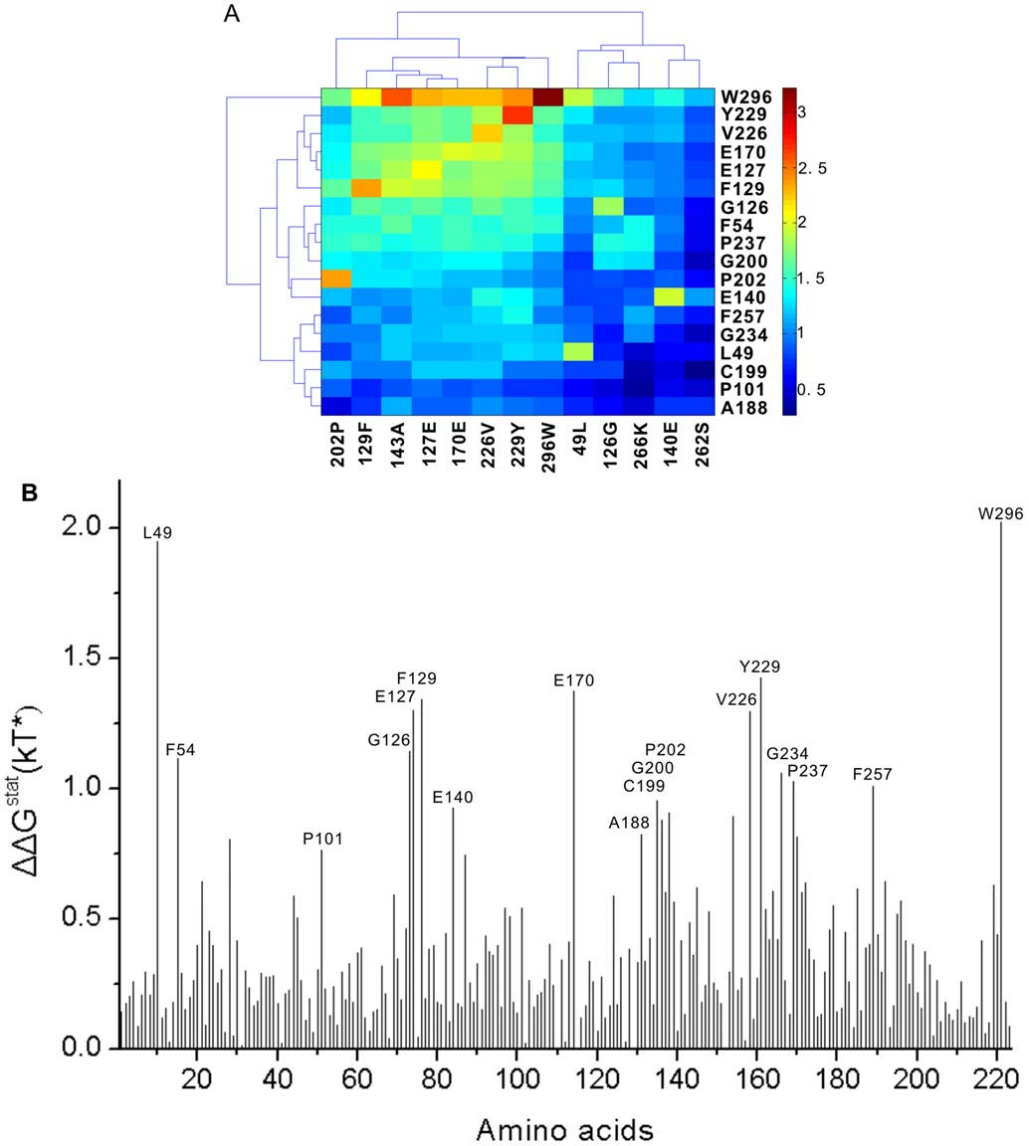
Clustering results of coupling energy matrix in the statistical coupling analysis (SCA). (A) The final cluster of 18 positions and 13 perturbations was obtained by iterative clustering. (B) The coupling energy profile of perturbation site 49L which is included in the final cluster.

To understand the information contained in this statistical coupling matrix, we performed an iterative two-dimensional clustering analysis. This analysis method was originally developed for identifying co-expressed gene clusters in many DNA microarray data analyses [33], [34]. The main idea is to narrow down both the perturbations that we use and the positions that are clustered, each time extracting the sub-matrix that were clustered together in the previous iteration and that contains large coupling energy or removing the sub-matrix that may disturb proper clustering results. Here, instead of clustering genes with similar expression profiles in different samples, we used iterative clustering to reveal positions in proteins that display similar patterns of statistical coupling in many perturbation experiments. Because these coupled “signals” may be masked by the “noises” generated by other positions, we checked each position and perturbation before clustering, and discarded 47 positions and 4 perturbations for which the standard deviation of their coupled values is less than 0.1 in corresponding rows or columns [34]. In general, only a small subunit of positions are strongly coupled, and these coupled positions should show self-consistency, that is, these positions couple largely only to each other [17], [18]. Two iterations of the clustering were performed (see Supporting Information Figure S4). In the first iteration, we found a cluster of 4 perturbations which have distinctly different coupling energy profiles and do not show the property of self-consistency. We filtered this cluster because it can interfere in the presentation of “proper position clusters”. In the second iteration, we focused on the matrix around regions of large ΔΔ*G^stat^* values, and identified a group of 18 positions and 13 perturbations that form a self-consistent cluster (Figure 2A). These positions show similar patterns of coupling for these 13 perturbations (see Supporting Information Figure S5) and all of them have high ΔΔ*G^stat^* values for any perturbation used to identify them (Figure 2B).

**Figure 3:**
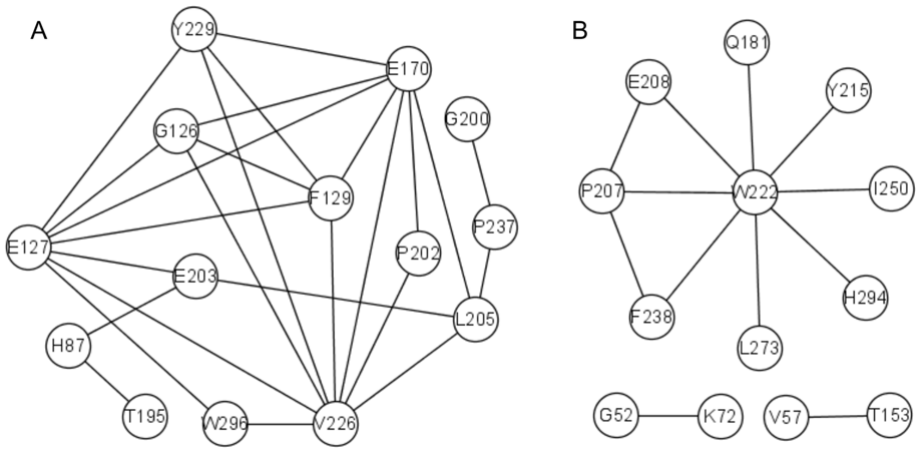
Results of mutual information (MI) analysis. (A) and (B) respectively denote one group of co-evolving positions. Linkages between positions in the MSA with residue numbers from a representative structure (1ATP) represent Z-scores more than 4.

**Figure 4:**
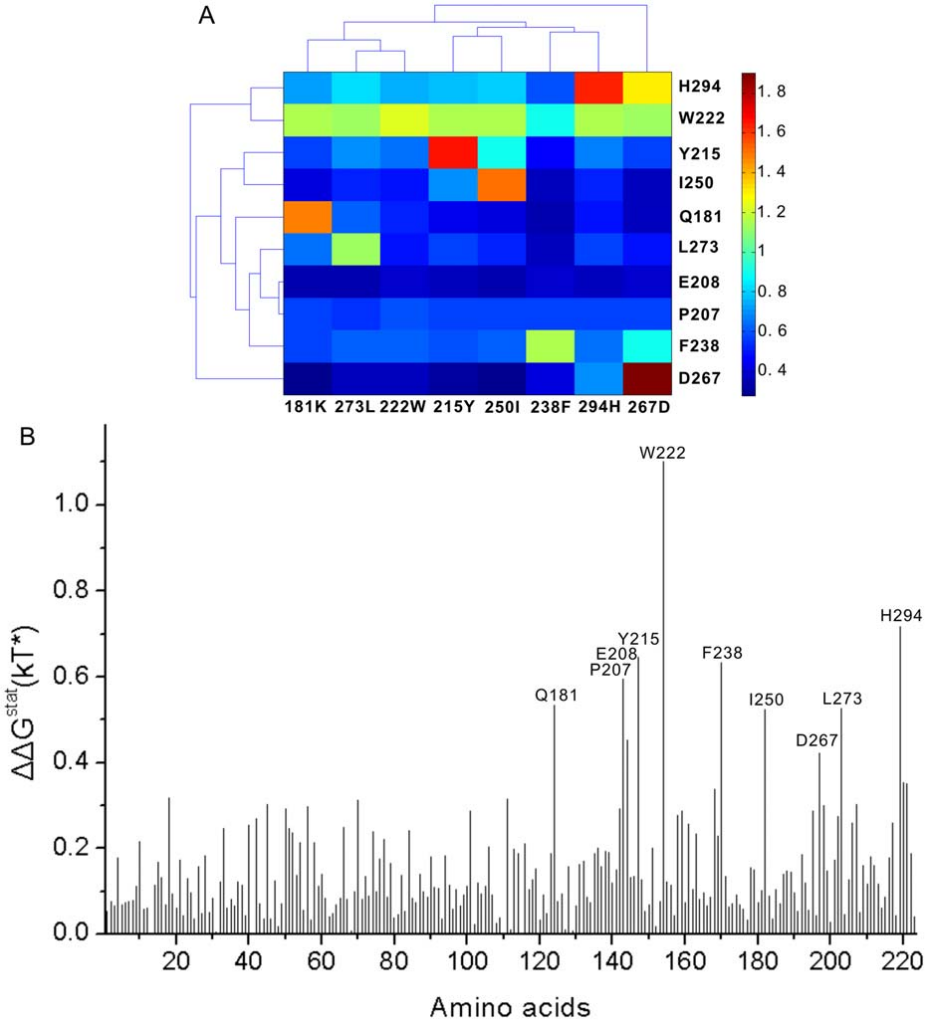
Clustering results of coupling energy matrix with perturbations obtained by MI analysis and SCA. (A) The final cluster of 10 positions and 8 perturbations was obtained by iterative clustering. (B) The coupling energy profile of perturbation site 222W which is included in the final cluser.

In order to validate the above results, we performed MI analysis with the same MSA except for two completely conserved positions. In MI analysis, we normalized each *MI* score by the corresponding pair entropy (*H_cd_*) to reduce the impact of phylogenetic correlations [35]. Similarly, we also removed the four exceptional sites identified in the above SCA in order to have proper results. The position pairs with the highest *MI*/*H_cd_* ratios were identified by calculating the Z-score for each ratio in each position pair. Prior modeling showed that a Z-score of 4 is the minimum value that reliably identified co-evolving positions in *in silico*-generated alignments [35], and so this Z-score value was chosen as our minimum level of significance. Unlike SCA, MI analysis identified two clusters of co-evolving residues as shown in Figure 3, and the positions included in Figure 3A have remarkable overlapping with those predicted by SCA. Apparently, a cluster of co-evolving residues was not found in the statistical coupling matrix by the clustering analysis. By rechecking the coupling energy profile of each perturbation site presented as a bar chart, we found the coupling energy profiles of several perturbations, as expected, are very similar (see Supporting Information Figure S6). After we extracted the coupling profiles of these perturbations to compose a new matrix for clustering (see Supporting Information Figure S7), we found a stable and self-consistent cluster of 10 positions, as shown in the Figure 4A. These positions remarkably overlap with sites in the Figure 3B predicted by MI analysis. Because the highest coupling energy values in these profiles (Figure 4B) are smaller than those of sites identified in Figure 2A and the number of sites in this coupled cluster is fewer, these coupled “signals” are liable to be masked and interfered by other higher “noises”, and as a result, the above clustering could not find out this coupled cluster.

In addition, we defined the highly conserved sites based on the probability of residues (p≥85%) at a site. There are 20 highly conserved sites in the MSA. After mapping these sites onto the 3D structural model of PKAc, we observed these highly conserved residues form a conserved core (see Supporting Information Figure S8). Most of them are hydrophobic amino acids. Apparently, such a hydrophobic core contributes to structural stability.

### Residue Correlation Analysis

**Figure 5:**
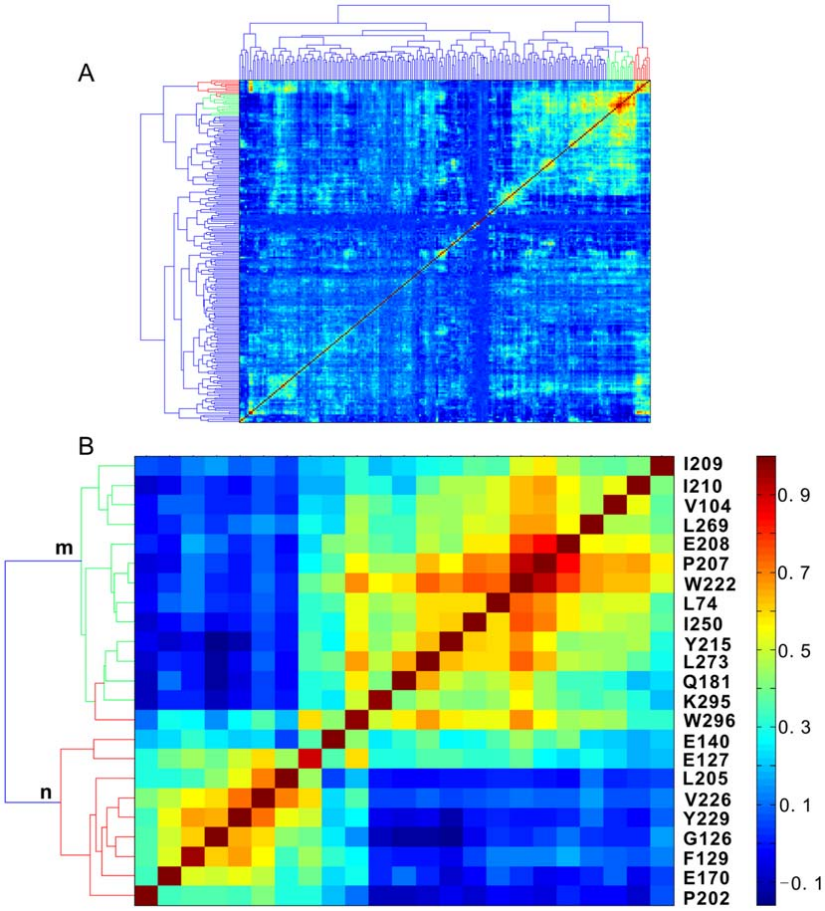
Results of residue correlation analysis (RCA). (A) Two-dimensional clustering analysis of correlation coefficients. The clusters with high correlation coefficients are highlighted by red and green lines in the dendrogram. (B) Closer view of the highly correlated clusters.

In order to further verify the above results, we performed RCA as described in [36] with the same MSA used in MI analysis. This analysis resulted in a 223×223 matrix. For the same purpose as in SCA, this matrix was clustered using the two-dimensional hierarchical clustering method (Figure 5). Two clusters of high correlation coefficients were indicated from the large background with the red and green lines in the dendrogram (Figure 5A). The details of these two clusters are shown in Figure 5B. The sites in clusters m and n substantially overlap with those in the corresponding clusters identified by both SCA and MI analysis, respectively. This demonstrated again the existence of two different clusters of correlated positions in the catalytic domains of Ser/Thr PKs.

**Figure 6:**
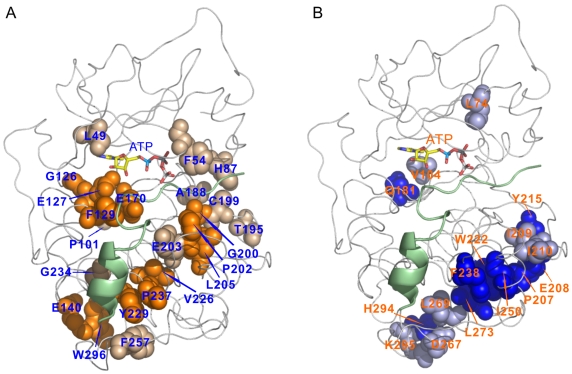
Mapping two clusters of correlated sites onto the 3D structure of PKAc. (A) The θ-shaped network. The orange sphere model shows the correlated sites identified by two or three methods, while the lightpink sphere model represents the correlated sites identified by only one method. (B) The γ-shaped network. The blue sphere model shows the correlated sites identified by two or three methods, while the lightblue sphere model represents the correlated sites identified by only one method. The peptide ligand is showed with lightgreen cartoon model. The stick model represents ATP molecule bound at active site cleft of PKA.

To compare these covariant positions identified by these three methods, we listed them in Table 1. Note that more than 70% of all positions predicted by one method are also identified by other one or two methods. To visualize these two different co-evolving position clusters, we mapped the positions of each cluster onto the 3D structure of PKAc (Figure 6). Most of these highly coupled positions in each cluster form a physically close network. According to the distribution of these positions on the 3D structure, we named these two networks as θ-shaped and γ-shaped networks, respectively (Figure 6A and 6B). These co-evolving positions can not be found by the structure or sequence conservation analysis.

**Table 1:** Highly coupled sites identified by SCA, RCA and MI analysis.

RCA	MI	SCA	RCA	MI	SCA	RCA	MI	SCA
		L49		T195		Y229	Y229	Y229
		F54			C199			G234
L74				G200	G200		P237	P237
	H87		P202	P202	P202		F238	F238
		P101		E203		I250	I250	I250
V104			L205	L205				F257
G126	G126	G126	P207	P207	P207			D267
E127	E127	E127	E208	E208	E208	L269		
F129	F129	F129	I209			L273	L273	L273
E140		E140	I210				H294	H294
E170	E170	E170	Y215	Y215	Y215	K295		
Q181	Q181	Q181	W222	W222	W222	W296	W296	W296
		A188	V226	V226	V226			

## Discussion

All members of Ser/Thr PK family have a common progenitor [8], [30]. As stated in the previous sections, the basic fold of catalytic domains of PKs is structurally well conserved. This conserved characteristic fold forms the structure basis of residue co-evolving in this family. Although these ancestral PKs have been heavily modified over the course of evolution to phosphorylate a variety of targets, interact with a range of partner proteins, and respond to different regulatory mechanisms, they might still share some common molecular mechanisms of both catalysis and substrate binding/releasing [30].

### The Θ-shaped Network

Most sites in the θ-shaped network are clustered in the substrate binding groove extending from the active site cleft to the C-terminus of the catalytic domain, some of which have been reported important for interactions with peptide substrates [4]. This fact indicates their importance in determining the substrate specificity of PKs. For instance, L49 and F54 lie in the nucleotide positioning motif and are flanked by highly conserved residues. G126, E127, F129, and E170 are situated in the linkage region, which links large and small lobes. The adenine ring and phosphates of ATP form many ionic and hydrogen-bonding interactions with many residues, including F54, E127 and E170 and other important residues [37], that are highly conserved and can not be detected by these covariant analysis methods [22]. E127 and E170 also interact with the P-3 and P-2 residues in the peptide substrate. G200 acts as the docking surface for the P site backbone. G202 and L205 have hydrophobic interactions with the P+1 residue [37], [38]. E203 provides a docking site for the P-6 residue. A recent study with a Ser/Thr PK, GSK-3β, indicated that the F67 (F54 in PKA) plays an important role in phosphorylating its substrate [39]. Several predicted substrate binding sites also interact with the conserved residues in the catalytic loop that in turn can align with the P-site hydroxyl group for transferring of the γ-phosphate group of ATP [40], [41]. Our results not only confirm these observations but also provide the details of the linking residues involved in this process.

It is now clear that in many cases short peptide substrate sequences may not exploit the complete binding capacity offered by PKs [42], and PKs may utilize one or more regions outside the consensus region for substrate recognition and recruitment [43], [44]. These additional docking interactions increase the affinity between kinases and their protein substrates by many times [45]. In Ser/Thr PKs, the docking domains are often part of the catalytic domain [7]. The strategic location of those distal residues away from the active site cleft in the θ-shaped network strongly suggests they might serve as docking or extended substrate-binding regions to participate in the interactions between PKs and their protein substrates. Most of these co-evolving residues are in an intermediate level of conservation so that many sites have not been noticed in previous studies [22]. Even so, crystal structures [46], [47] and mutagenesis [48] studies have provided some support for this speculation. In different kinase subfamilies, these sites may have different residues [3], [4], and different substrates recognize different positions [46], [47]. Differences in the composition of residues in the distal sites and the local preferences of the catalytic core for different amino acids around the P-site in substrates work together to increase the overall selectivity of kinase-substrate interactions [49].

Through interactions of co-evolving residues, protein substrate binding induces conformational changes of PKs that are essential for phosphoryl transfer [50]–[52]. In the opposite direction, ATP binding, situated in the active site cleft, also induces conformational changes and is coupled with distal substrate binding [50], [52]–[54]. From the distribution of these coupled sites, we believe the θ-shaped network may provide a structural basis for this coupling [55]. Recently, Taylor's group proposed that there appear to be two lines of communication in coupling adenosine binding with peptide binding [56]. One is from the active site cleft to the D helix via E127; and the second possible link from the active site cleft to peptide-binding ledge is through the F helix in two ways. One way is that the F helix may communicate ATP binding from highly conserved D220 to substrate binding regions. These two links are identical with the θ-shaped network predicted by us. Both V226 and Y229 predicted by us are exactly situated in the F helix. A second way is through F238 and W222. This link is overlapping with the γ-shaped network predicted here, which will be discussed later. In a word, the θ-shaped network may play important roles in PKs interacting with their substrates, which matches some previous experimental results.

### The Γ-shaped Network

In the γ-shaped network, the coupled residues, mainly clustered in one side of the substrate binding regions, link the activation loop with different parts of the substrate binding regions. Several publications provided important implications for the roles of this network. Crystal structures implicated that this network may have functions in stabilizing the activation loop and substrate binding regions [41], [56]. However, recent studies with *S. cerevisiae* PKA mutants Tpk1^K336A/H338A^ and Tpk1^R324A^, mutant residues corresponding to the K292, H294, R280 in PKA, found these mutants can bind more of their substrates than the wild protein in *S. cerevisiae*, and the binding observed was specific to substrates and was dependent upon residues known to be important for interactions with peptide substrates [48]. H294 is within the γ-shaped network. R280 is a highly conserved site and forms an ion pair with another conserved site E208, which is also included in the γ-shaped network. This ion pair is stabilized by another site in the γ-shaped network, W222 [37]. W222 may play a key role in substrate-associated conformational changes [57]. These observations suggest that the γ-shaped network might participate in the phosphoproduct releasing, and the structure stability is important for this network to perform this function. In the CK1 subfamily of Ser/Thr PKs, the A206-P207-E208 sequence is replaced by the motif SIN, which is conserved in the CK1 subfamily, hence the ion pair (E208-R280) is not formed, but residue E202 (W222) forms a new ion pair with residue R261 (L273 in PKA) [30]. The overall structure of the C-terminal sub-domain of CK1 is still very similar with those for other Ser/Thr PKs. Coincided with the phosphoryl transfer step, an order-disorder transition was generated due to an internal entropy contribution to catalysis [58]. The electrostatic repel between the highly conserved site D166 and the phospho-residue in product could serve to facilitate the phosphoproduct dissociation [59]. Based on all these results, we deduce that the γ-shaped network stabilizes the activation loop and substrate binding regions before catalysis, and after phosphoryl transfer, serves as a signaling pathway by which the electrostatic repel energy and the local conformational changes due to internal entropy contribution are propagated and lead to the release of phosphoproduct.

In the end, there are several issues to be concerned with. In SCA, we found that four exceptional perturbations can generate remarkably high coupling energy for many other sites, but these sites are not self-consistent (see Supporting Information Figure S9A). We mapped these four sites onto the 3D structure of PKAc and found that these sites are situated on the boundary between these two networks (Figure S9B). Open question remains as to what roles these sites play in proteins. In addition, it has been noted that site pairs identified as correlated tend to be relatively close in protein tertiary structure, however, many authors have proved that correlations between sites that are physically distant in protein structures might also be attributable to protein function [16], [31], [60]–[62]. We do not know how some coupled sites, such as Leu74, Val104 and Gln181, which are physically distant from other sites, are functionally correlated with other residues (Figure 6B). In general, these co-evolving positions are thermodynamically coupled, but there is little evidence for the hypothesis that thermodynamic coupling is limited to the subset of co-evolving residues. Consequently, our results only demonstrate that we identified two distinct co-evolving networks in the catalytic domains of Ser/Thr PKs by using correlated mutation analysis, and these two networks might play important roles in mediating PKs interacting with their substrates. We do not deny that other residues might also participate in such a process directly or indirectly. Our results do not provide more details on how these coupled residues play their roles in the process of PKs interacting with their substrates. However, our results have been able to provide new helpful information for further understanding the mechanisms of this interacting process.

## Materials and Methods

### Data Source and Multiple Sequence Alignment

The sequences of the catalytic domains of Ser/Thr PKs were collected from the non-redundant database of protein sequences by PSI-BLAST [63] with default settings; seventeen representative eukaryotic catalytic domain sequences with known structures were used in initial searches, of which fifteen was included in the HOMSTRAD [64]. The structure-based MSA was created by using FUGUE [29]. Alignments were then manually adjusted to improve the overall alignment. Because cAMP-dependent Protein kinase (PKA) often serves as a prototype of the entire PKs [37], the final alignment was truncated to include only positions present in the catalytic subunit of PKA (PKAc), and for all calculations, the numbering of PKAc was used. Finally, the alignment of 1238 sequences including 347 sites is available for analysis.

### Statistical Coupling Analysis (SCA)

The static energy (Δ*G^stat^*) for each site and statistical coupling energy (ΔΔ*G^stat^*) between any two sites were calculated by using the SCA method described by Lockless and Ranganathan [14] and Fodor *et al*. [65]. The programs were written in MATLAB (Mathworks).

### Mutual Information (MI) Analysis

MI analysis was performed according to the methods described in [35]. To reduce the influence of entropy on *MI* values, the raw *MI* values were normalized (*i.e.* divided by the joint entropy of the corresponding positions, *H_cd_*). A Z-score (the number of standard deviations of *MI/H_cd_*) was assigned to each normalized ratio. If a Z-score was above a fixed threshold ( = 4), two corresponding sites were linked by an edge, and each site was represented as a node. The calculation programs were written in MATLAB. Network graphs were visualized in Cytoscape 2.0 [66].

### Residue Correlation Analysis (RCA)

Correlated coefficients for any two sites were calculated as previously described [11], [36]. For a given pair of sequences (*k*, *l*), each substitution at a position (*i* or *j*) is associated with a similarity score (*X_ikl_* or *X_jkl_*, respectively) obtained from the McLachlan scoring matrix [67]. The program was written in Fortran.

### Clustering Analysis

As for SCA, a two-dimensional hierarchical clustering analysis was iteratively performed on coupling energy matrices in order to identify co-evolving residues in the catalytic domains of Ser/Thr PKs [33]. The Euclidean distance was used for calculating distances, and the complete linkage was used in clustering. As for RCA, a similar two-dimensional hierarchical clustering analysis was carried out on a correlation coefficient matrix, but did not need iterative clustering. Softwares were written in MATLAB.

## Supporting Information

Text S1 This file includes the multiple sequence alignment of catalytical domains of Ser/Thr protein kinases.
(0.25 MB TXT)

Figure S1 The distribution of initial query sequences which were used to collect the homologue sequences on the phylogenetic tree of eukaryotic PK family. The phylogenetic tree is visualized by HyperTree sofeware
(1.20 MB TIF)

Figure S2 A colorful representation for the partial result of sequence alignment with the representative protein structures of catalytic domains of serine/threonine kinases family (top 15 lines) using FUGUE
(2.00 MB TIF)

Figure S3 Statistical equilibrium in MSA and criterion for selection of perturbations. (A) The average static energy at ten unconserved sites is plotted against the number of sequences randomly selected from the complete MSA. (B) The average statistical coupling energy for ten unconserved sites is plotted against the number of sequences randomly selected from the complete MSA. This plot is for perturbation site 170. Other perturbations can be tested according to the same method. Error bars represent the standard deviation of the mean at the ten sites.
(0.78 MB TIF)

Figure S4 Iterative clustering of the statistical coupled matrix for the catalytic domains family of Ser/Thr PKs. (A) The unclustered matrix, ordered by positions (N to C terminus) on rows perturbations (N to C terminus) on columns. (B) The initial round of two-dimensional hierarchical clustering revealed one cluster of perturbations has distinct coupling energy profile, and is not self-consistent. These perturbations were represented by magenta lines in the dendrogram. Self-consistency means that each cluster represents a set of positions that couple largely only to each other. (C) The next round involved removing the perturbation cluster which is not self-consistent, and then re-clustering. This round revealed one cluster which is self-consistent and it was represented by red lines in the dendrogram. (D) At the final round, the sub-matrix corresponding to the red lines from (C) was extracted and re-clustered
(1.35 MB TIF)

Figure S5 Two profiles of statistical coupling energy generated by perturbing at residues 49 (A) and 170 (B) included within the θ-shaped network are shown in order to explain the self-consistency of this network.
(1.20 MB TIF)

Figure S6 Two profiles of statistical coupling energy generated by perturbing at residues 222 (A) and 294 (B) are represented to illustrate the self-consistency of these sites.
(1.00 MB TIF)

Figure S7 Iterative clustering of the statistical coupling matrix in order to identify another cluster of coupled positions. (A) The unclustered matrix as illustrated in Figure S4. (B) The initial round of two-dimensional hierarchical clustering with a group of specific perturbations chosen by observing the results of MI and SCA invealed a group of positions which are self-consistent and they were represented by the red lines in the dendrogram. (C) The sub-matrix corresponding to the red lines from (B) was extracted and reclustered
(0.76 MB TIF)

Figure S8 Mapping only the highly conserved sites (A) and mapping both the highly conserved and co-evolving sites identified by SCA (B) onto the tertiary structure of PKA catalytic domain (1ATP).
(1.35 MB TIF)

Figure S9 Mapping four exceptional sites onto the tertiary structure of 1ATP. (A) The result of initial round clustering as demonstrated in Figure S2B. The magenta lines on the column represent four exceptional sites that have distinct coupling energy profiles. (B) These four sites are mapped onto the 3D structure of 1ATP. (C) Showing the relationship between these exceptional sites and two co-evolving networks on the 3D structure of 1ATP.
(1.88 MB TIF)
