# Correction: Correlated Mutation Analysis on the Catalytic Domains of Serine/Threonine Protein Kinases

**DOI:** 10.1371/annotation/654a1794-3ca1-45ac-bbf6-20ae9d33c016

**Published:** 2009-06-24

**Authors:** Feng Xu, Pan Du, Hongbo Shen, Hairong Hu, Qi Wu, Jun Xie, Long Yu

An institutional affiliation of the 7th author, Long Yu, was omitted. Affiliation number 1, State Key Laboratory of Genetic Engineering, Institute of Genetics, School of Life Sciences, Fudan University, Shanghai, China, should be added for this author.

