# Supplementary figures and images for: Correlated Mutation Analysis on the Catalytic Domains of Serine/Threonine Protein Kinases
Source: PLoS One. 2009 Jun 15;4(6):e5913. doi: 10.1371/journal.pone.0005913 (PMC2690836; doi:10.1371/journal.pone.0005913)

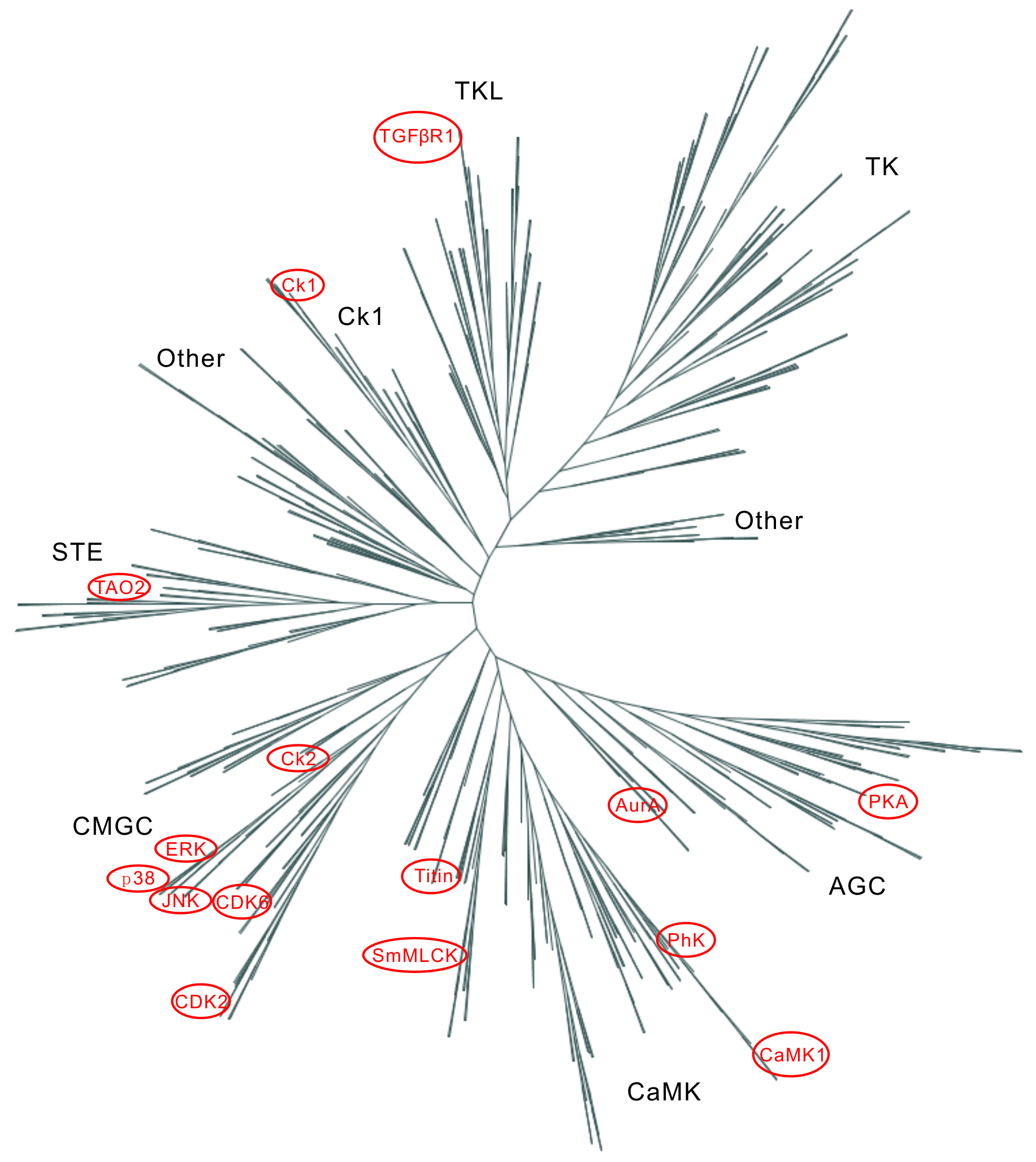

Supplement: Figure S1 — The distribution of initial query sequences which were used to collect the homologue sequences on the phylogenetic tree of eukaryotic PK family. The phylogenetic tree is visualized by HyperTree sofeware (1.20 MB TIF) [file pone.0005913.s002.tif]

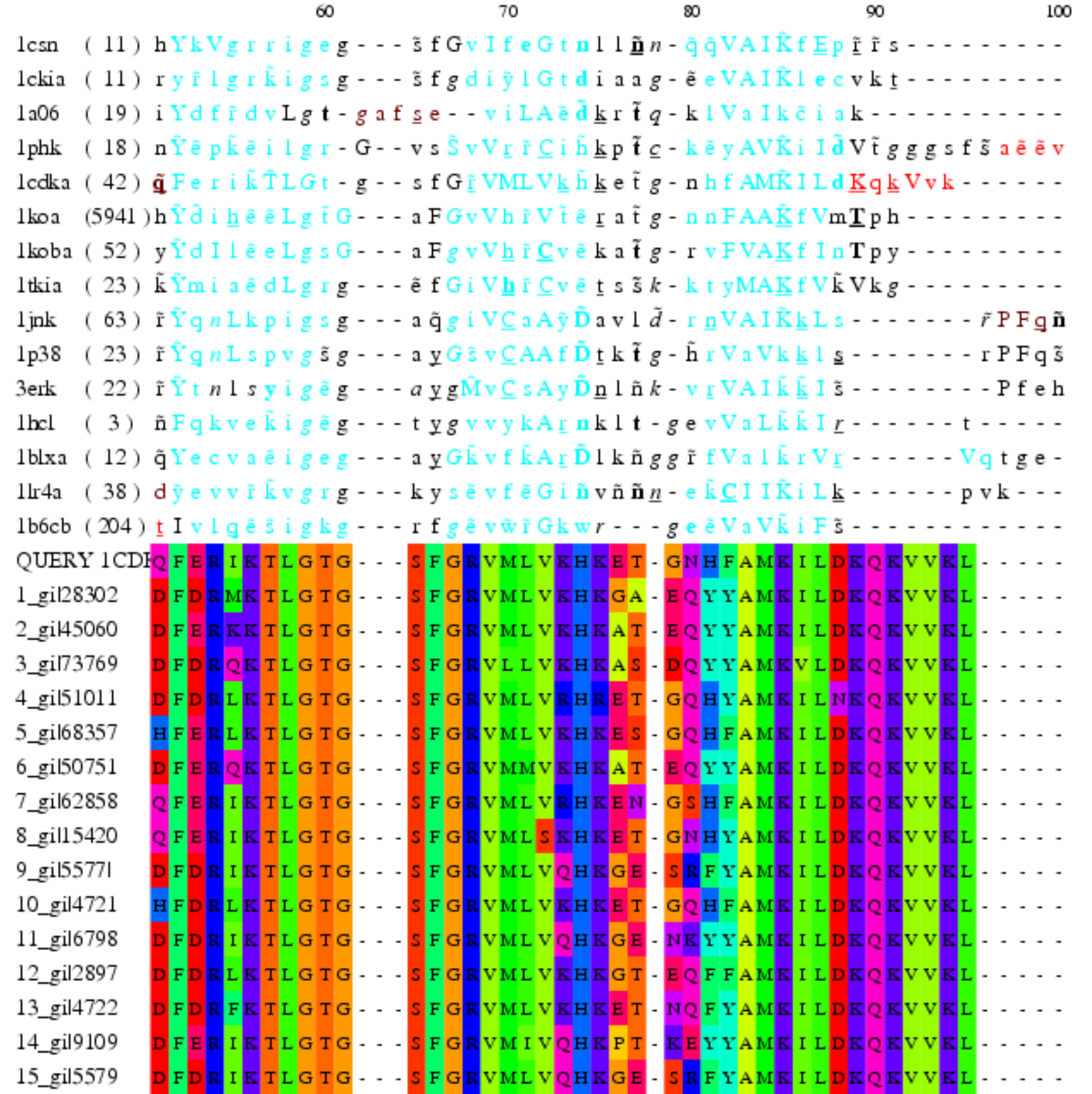

Supplement: Figure S2 — A colorful representation for the partial result of sequence alignment with the representative protein structures of catalytic domains of serine/threonine kinases family (top 15 lines) using FUGUE (2.00 MB TIF) [file pone.0005913.s003.tif]

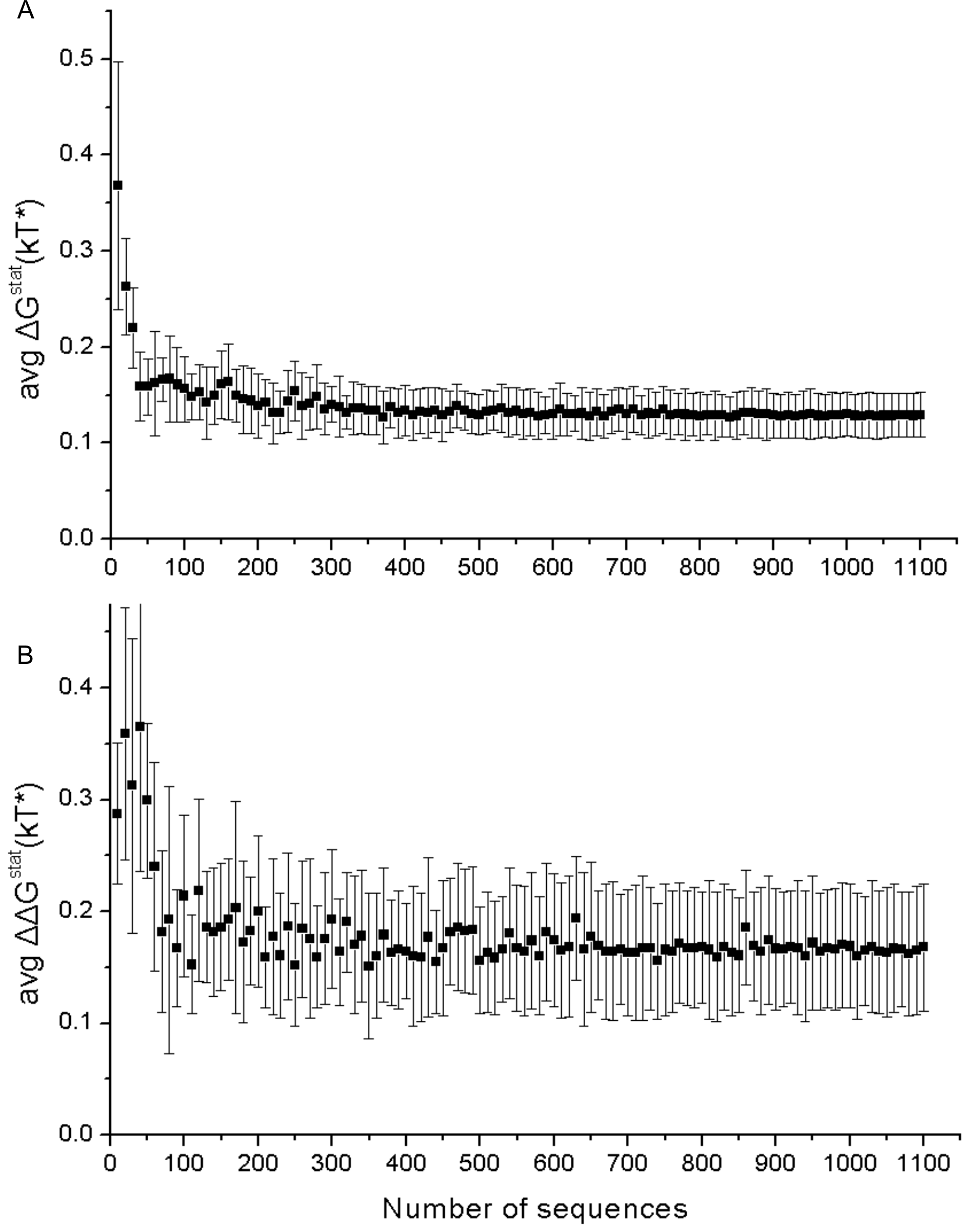

Supplement: Figure S3 — Statistical equilibrium in MSA and criterion for selection of perturbations. (A) The average static energy at ten unconserved sites is plotted against the number of sequences randomly selected from the complete MSA. (B) The average statistical coupling energy for ten unconserved sites is plotted against the number of sequences randomly selected from the complete MSA. This plot is for perturbation site 170. Other perturbations can be tested according to the same method. Error bars represent the standard deviation of the mean at the ten sites. (0.78 MB TIF) [file pone.0005913.s004.tif]

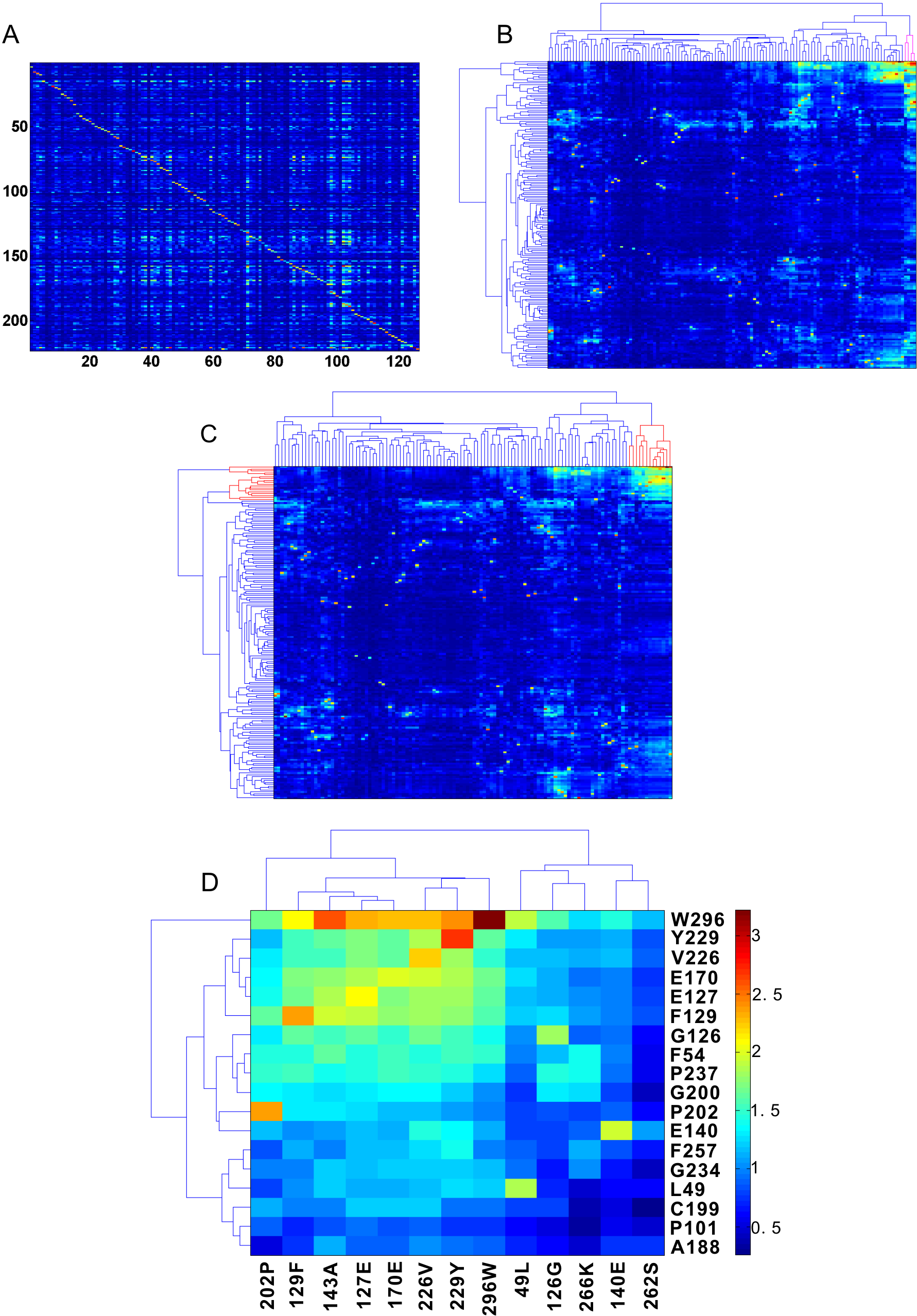

Supplement: Figure S4 — Iterative clustering of the statistical coupled matrix for the catalytic domains family of Ser/Thr PKs. (A) The unclustered matrix, ordered by positions (N to C terminus) on rows perturbations (N to C terminus) on columns. (B) The initial round of two-dimensional hierarchical clustering revealed one cluster of perturbations has distinct coupling energy profile, and is not self-consistent. These perturbations were represented by magenta lines in the dendrogram. Self-consistency means that each cluster represents a set of positions that couple largely only to each other. (C) The next round involved removing the perturbation cluster which is not self-consistent, and then re-clustering. This round revealed one cluster which is self-consistent and it was represented by red lines in the dendrogram. (D) At the final round, the sub-matrix corresponding to the red lines from (C) was extracted and re-clustered (1.35 MB TIF) [file pone.0005913.s005.tif]

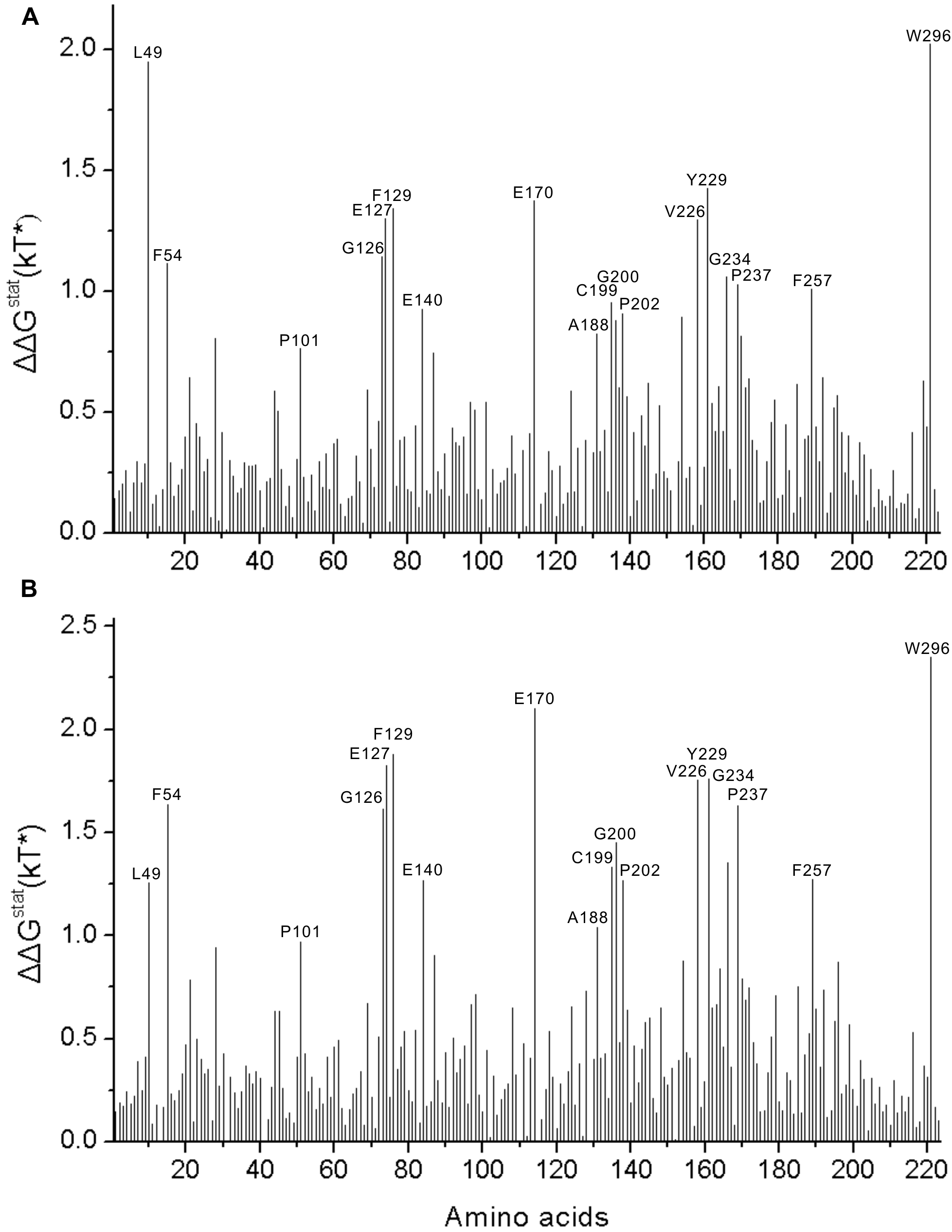

Supplement: Figure S5 — Two profiles of statistical coupling energy generated by perturbing at residues 49 (A) and 170 (B) included within the θ-shaped network are shown in order to explain the self-consistency of this network. (1.20 MB TIF) [file pone.0005913.s006.tif]

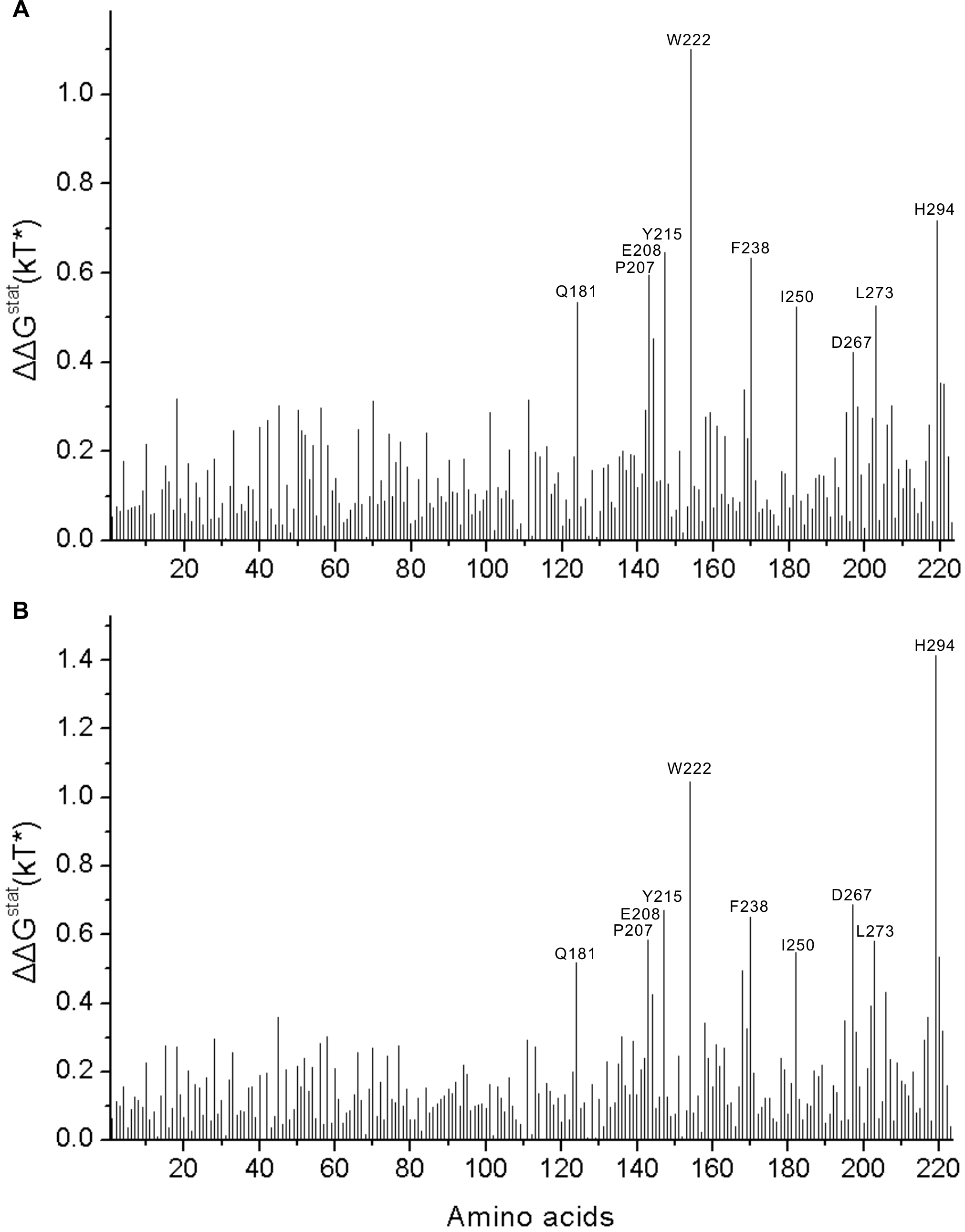

Supplement: Figure S6 — Two profiles of statistical coupling energy generated by perturbing at residues 222 (A) and 294 (B) are represented to illustrate the self-consistency of these sites. (1.00 MB TIF) [file pone.0005913.s007.tif]

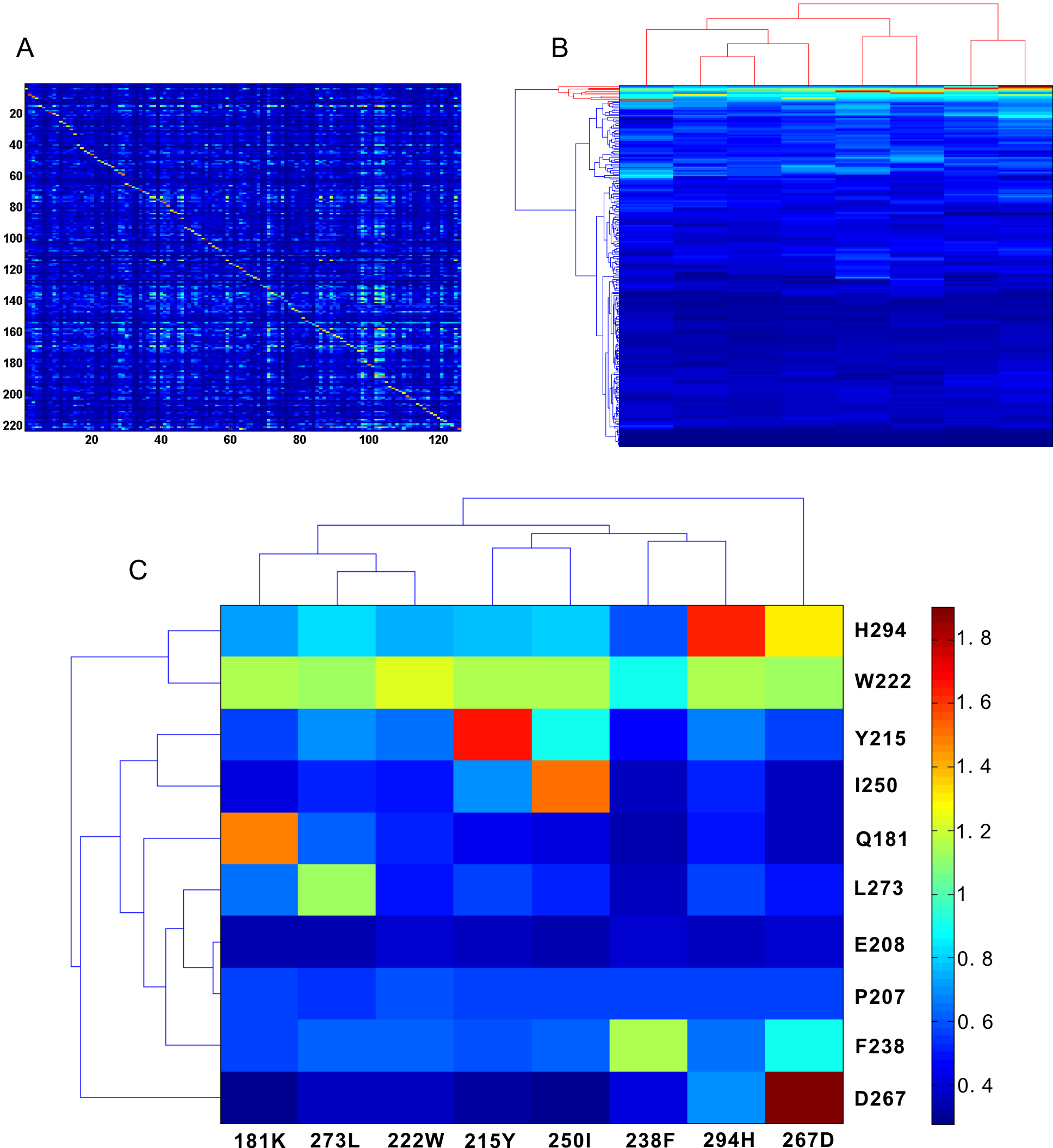

Supplement: Figure S7 — Iterative clustering of the statistical coupling matrix in order to identify another cluster of coupled positions. (A) The unclustered matrix as illustrated in Figure S4. (B) The initial round of two-dimensional hierarchical clustering with a group of specific perturbations chosen by observing the results of MI and SCA invealed a group of positions which are self-consistent and they were represented by the red lines in the dendrogram. (C) The sub-matrix corresponding to the red lines from (B) was extracted and reclustered (0.76 MB TIF) [file pone.0005913.s008.tif]

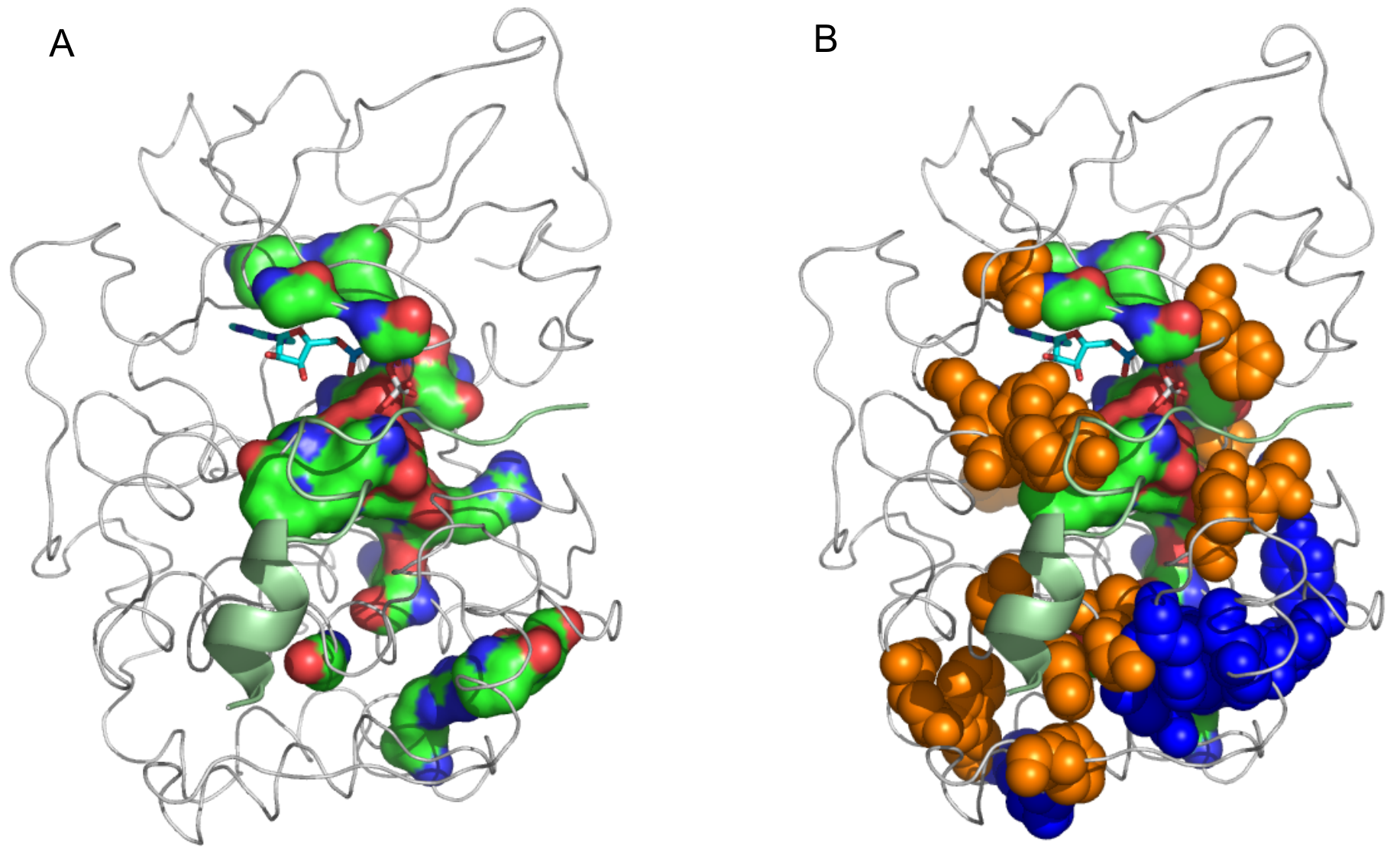

Supplement: Figure S8 — Mapping only the highly conserved sites (A) and mapping both the highly conserved and co-evolving sites identified by SCA (B) onto the tertiary structure of PKA catalytic domain (1ATP). (1.35 MB TIF) [file pone.0005913.s009.tif]

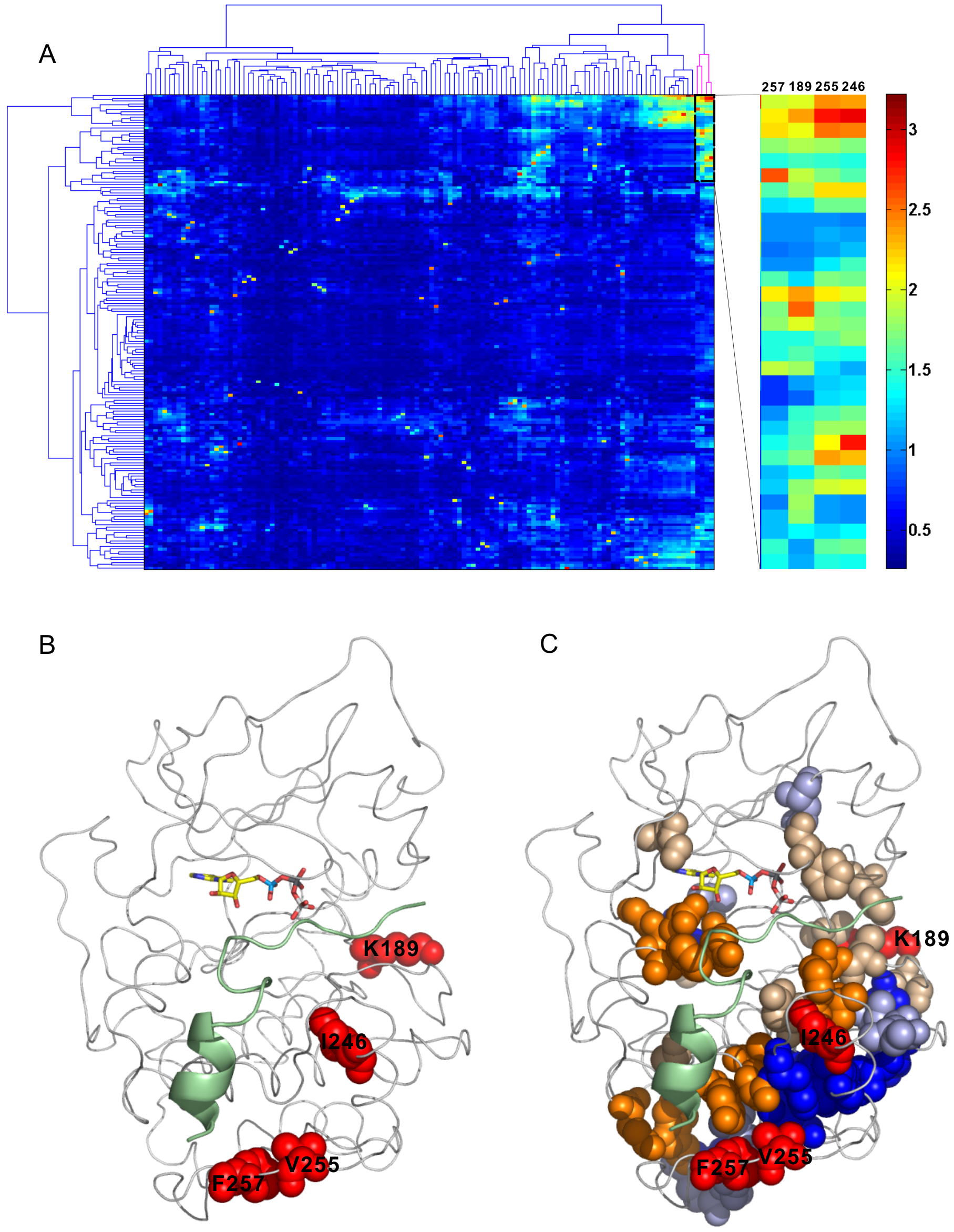

Supplement: Figure S9 — Mapping four exceptional sites onto the tertiary structure of 1ATP. (A) The result of initial round clustering as demonstrated in Figure S2B. The magenta lines on the column represent four exceptional sites that have distinct coupling energy profiles. (B) These four sites are mapped onto the 3D structure of 1ATP. (C) Showing the relationship between these exceptional sites and two co-evolving networks on the 3D structure of 1ATP. (1.88 MB TIF) [file pone.0005913.s010.tif]
